# Lack of Associations between* XPC* Gene Polymorphisms and Neuroblastoma Susceptibility in a Chinese Population

**DOI:** 10.1155/2016/2932049

**Published:** 2016-10-26

**Authors:** Jintao Zheng, Ruizhong Zhang, Jinhong Zhu, Fenghua Wang, Tianyou Yang, Jing He, Huimin Xia

**Affiliations:** ^1^Department of Pediatric Surgery, Foshan Maternity and Children's Healthcare Hospital Affiliated to Southern Medical University, Foshan, China; ^2^Department of Pediatric Surgery, Guangzhou Institute of Pediatrics, Guangzhou Women and Children's Medical Center, Guangzhou Medical University, Guangzhou, Guangdong 510623, China; ^3^Molecular Epidemiology Laboratory and Department of Laboratory Medicine, Harbin Medical University Cancer Hospital, Harbin, Heilongjiang 150040, China

## Abstract

Neuroblastoma is one of the most malignant solid tumors in infants and young children. No more than 40% of neuroblastoma patients can survive for longer than five years after it has been diagnosed. XPC protein is a pivotal factor that recognizes DNA damage and starts up the nucleotide excision repair (NER) in mammalian cells. This makes up the first group to defend against the cancer. Previous studies have identified that* XPC* gene polymorphisms were associated with various types of cancer. However, the associations between* XPC* gene polymorphisms and neuroblastoma risk have not yet been studied. We investigated the associations between three* XPC* gene polymorphisms (rs2228001 A>C, rs2228000 C>T, and rs2229090 G>C) and neuroblastoma risk with 256 neuroblastoma patients and 531 healthy controls in a Chinese Han population. Odds ratios and 95% confidence intervals were used to access the association between these three polymorphisms and neuroblastoma risk. No significant association was detected between these three polymorphisms and neuroblastoma risk in the overall analysis as well as in the stratification analysis. These results suggest that none of these three polymorphisms may be associated with the risk of neuroblastoma in the Chinese Han population.

## 1. Introduction

Neuroblastoma originates in primitive neural crest cells of the adrenal medulla or sympathetic ganglia. It is one of the most malignant solid tumors in infants and young children, in particular, accounting for 7%−10% of childhood tumors. The tumor is found to primarily locate in the retroperitoneal parts (approximately 60%) and secondly in the mediastinum, pelvis, and cervical sympathetic ganglion. The rest, about 12%, associates with other malformations [1–3]. The morbidity of neuroblastoma in the live births is about 7.7 cases per million in China [4]. Despite the application of multimodality treatment including surgery, chemotherapy, and radiotherapy, no more than 40% of neuroblastoma patients could survive for longer than five years after diagnosis. Until now, the etiology of neuroblastoma remains largely unclear [5, 6].

Genome-wide association study (GWAS) has been used as a convenient and powerful approach for examining inherited genetic variations in relation to human complex disease, such as cancer susceptibility [7, 8]. In 2008, Maris et al. [9] used the GWAS approach to prove that three single nucleotide polymorphisms (SNPs) in* CASC15* gene at chromosome 6p22 (rs6939340, rs4712653, and rs9295536) were highly associated with neuroblastoma susceptibility in European descents. Since then, lots of SNPs in specific chromosomal regions have been identified by the GWAS approach. Several gene polymorphisms are significantly associated with the risk of neuroblastoma, such as* LIN28B* [10],* HACE1* [10],* BARD1* [11],* LMO1* [12],* HSD17B12* [13],* DDX4* [13],* IL31RA* [13], and* DUSP12* [13], which have been discovered by means of GWAS. Besides, several genes, such as* FAS* [14],* FASL* [14],* NEFL* [15], and* TGFBR3L* [16], have been discovered by candidate gene approach.

Nucleotide excision repair (NER) can preserve the hereditary integrity and stability of genomic DNA through eliminating DNA damage generated by exogenous agents (i.e., mutagenic and carcinogenic substances) and photoproducts caused by sunlight exposure [17, 18]. In humans, hereditary defects of genomic DNA in NER are involved in several autosomal recessive diseases. Xeroderma pigmentosum (XP) is characterized by a strong predisposition to skin carcinomas. However, it was not until 1968 that Cleaver [19] identified that XP could result from the defects in NER. NER is a complex biochemical process that involves hundreds of components in human cells, including the seven XP-related genes (*XPA*,* XPB*,* XPC*,* XPD*,* XPE*,* XPF*, and* XPG*). XPC protein is an important DNA damage recognition protein and an initiator of the NER process that defends against cancer [20–25].

Some* XPC* gene SNPs have been identified to be implicated in melanoma [26], colorectal cancer [27, 28], prostate cancer [29], hepatocellular cancer [30], lung cancer [31], and gastric cancer [32]. However, the association between* XPC* gene polymorphisms and neuroblastoma risk has not been studied. In view of this, we investigated the relationship between* XPC* gene polymorphisms (rs2228001 A>C, rs2228000 C>T, and rs2229090 G>C) and neuroblastoma susceptibility in a Chinese Han population making use of a total of 787 participants (256 cases and 531 controls).

## 2. Materials and Methods

### 2.1. Study Subjects

A total of 256 newly diagnosed and histopathologically confirmed neuroblastoma cases were recruited from the Guangzhou Women and Children's Medical Center between February 2010 and November 2015 [33–35]. During the same period, 531 healthy children were randomly selected as the age- and gender-matched controls after receiving routine physical examination [35–37]. Both the cases and the controls were unassociated ethnic Chinese Han individuals. The study was granted permission by the Institutional Review Board of Guangzhou Women and Children's Medical Center. Demographic factors and medical histories were gathered through the structured questionnaires. Written informed consent was received from the guardians of each child participant. Each participant donated 2 mL of blood for genomic DNA extraction.

### 2.2. Polymorphism Analysis

The selection criteria were described previously, in terms of the minor allele frequency, putative functional potentials, and low linkage disequilibrium [38]. Based on the criteria, three potentially functional SNPs (rs2228001 A>C, rs2228000 C>T, and rs2229090 G>C) were selected. s2228001 A>C (Lys939Gln) and rs2228000 C>T (Val499Arg) are nonsynonymous SNPs and they have been widely investigated in various types of cancer.* XPC* rs2229090 G>C is 3′UTR polymorphism within miRNA binding site. Genomic DNA was extracted from 2 mL of peripheral blood sample using the TIANamp Blood DNA Kit (TianGen Biotech Co. Ltd., Beijing, China) following the manufacturer's instructions. Qualified DNA samples were diluted to 10 ng/*μ*L and loaded in 96-well plates. Then, these three SNPs were genotyped using Taqman real-time PCR method as described previously [39, 40].

### 2.3. Statistical Analysis

*χ* ^2^ test was used to evaluate the differences in the frequency distributions of the demographics and genotypes between the neuroblastoma cases and the controls. Hardy-Weinberg equilibrium (HWE) was tested in the controls by the goodness-of-fit chi-squared test. Odds ratios (ORs) and 95% confidence intervals (CIs) were used to assess the correlations between the three polymorphisms and neuroblastoma susceptibility with the unconditional multivariate logistic regression analysis. *P* value < 0.05 was considered as statistically significant. All statistical tests were two-sided and analyzed using SAS software (version 9.1; SAS Institute, Cary, NC).

## 3. Results

### 3.1. Frequency Distribution of Selected Characteristics

As displayed in Supplemental Table  1 in Supplementary Material available online at http://dx.doi.org/10.1155/2016/2932049, the average age of the cases was 30.87 ± 26.45 months and 29.73 ± 24.86 months for the controls. No significant differences were observed in terms of age (*P* = 0.239) and gender (*P* = 0.333) between the case and the control groups. According to the INSS standard [3], 54, 65, 44, 77, and 9 patients developed clinical stages I, II, III, and IV and 4s neuroblastoma, respectively. Among these cases, 46 lesions occurred in adrenal gland, 87 in retroperitoneal region, and 90 in mediastinum.

### 3.2. *XPC* Gene Polymorphisms and Neuroblastoma Susceptibility

In the current study, 253 cases and 531 controls were successfully genotyped. The genotype frequencies of the three polymorphisms are shown in Table 1. The observed genotype frequencies of the three SNPs were in accordance with HWE in the control subjects (*P* = 0.948 for rs2228001 A>C polymorphism, *P* = 0.988 for rs2228000 C>T polymorphism, and *P* = 0.994 for rs2229090 G>C polymorphism). There is no significant association between rs2228001 A>C polymorphism and neuroblastoma susceptibility. Similar results were found for rs2228000 C>T and rs2229090 G>C polymorphisms.

### 3.3. Stratification Analysis of* XPC* Gene Polymorphisms with Neuroblastoma Susceptibility

Stratified analyses were conducted regarding age, gender, sites of origin, and clinical stages to assess the association of the three selected polymorphisms with the risk of neuroblastoma (Table 2). However, no significant association was identified for any of the three polymorphisms.

## 4. Discussion

In this hospital-based study comprising 256 cases and 531 controls, none of the three* XPC* gene polymorphisms was associated with neuroblastoma risk when compared to the reference genotypes. To the best of our knowledge, this is the first investigation looking into the relationship between* XPC* gene polymorphisms and neuroblastoma susceptibility in a Chinese Han population.

*XPC* gene (http://www.ncbi.nlm.nih.gov/gene/7508) is located on chromosome 3p25.1 with 18 exons, which encodes a component of the NER. XPC plays a distinctively vital role in the early stages of global genome NER. XPC and UV excision repair protein RAD23 homolog B (HR23B) form the XPC-HR23B complex, which recognizes DNA damage and initiates NER in mammalian cells, thereby protecting against cancer [20–25, 41, 42].

*XPC* gene polymorphisms are involved in the different types of cancer. Among them, rs2228001 A>C and rs2228000 C>T were widely investigated. Paszkowska-Szczur et al. [26] genotyped 714 melanoma cases and 1841 healthy controls to evaluate the relationship between 94 SNPs within the seven* XP* genes (*XPA*–*XPG*) and the melanoma risk in a Polish population. They found that* XPC* rs2228000 CT and TT genotypes were significantly associated with decreased melanoma risk when compared with the reference genotype. Moreover, they observed that rs2228000 CT genotype and rs2228000 TT genotype were associated with decreased colorectal cancer risk compared to the CC genotype in 758 colorectal cancer patients and the same number of controls [27]. In a study conducted in Malaysia with 255 colorectal cancer patients and 255 controls, Ahmad Aizat et al. [28] found that XPC gene rs2228000 GG genotype was associated with an increased colorectal cancer risk. However, there is no previous study investigating the association between* XPC* gene polymorphisms and neuroblastoma susceptibility [43]. Our study fits the niche and it found no association between the three SNPs of* XPC* gene and neuroblastoma. However, the negative results might result from the relatively small sample size in this study, although it was the largest study regarding the Chinese children to date.

## 5. Conclusions

In summary, all the three* XPC* gene polymorphisms (rs2228001 A>C, rs2228000 C>T, and rs2229090 G>C) may not associate with neuroblastoma risk in the Chinese Han population. However, further studies with larger sample size and different ethnicities should be carried out to verify our results.

## Supplementary Material

Supplemental Table 1: Frequency distribution of selected characteristics for neuroblastoma cases and cancer-free controls.

## Figures and Tables

**Table 1:** Genotype and allele frequencies of the three selected polymorphisms and neuroblastoma susceptibility in a Chinese population.

Genotype	Cases (*N* = 253)	Controls (*N* = 531)	*P* ^a^	Crude OR (95% CI)	*P*	Adjusted OR (95% CI)^b^	*P* ^b^
rs2228001 (HWE = 0.948)							
AA	99 (39.13)	218 (41.05)		1.00		1.00	
AC	118 (46.64)	245 (46.14)		1.06 (0.77–1.47)	0.722	1.05 (0.76–1.45)	0.769
CC	36 (14.23)	68 (12.81)		1.17 (0.73–1.86)	0.521	1.15 (0.72–1.84)	0.555
Additive			0.807	1.08 (0.86–1.34)	0.520	1.07 (0.86–1.33)	0.563
Dominant	154 (60.87)	313 (58.95)	0.608	1.08 (0.80–1.47)	0.608	1.07 (0.79–1.46)	0.656
Recessive	217 (85.77)	463 (87.19)	0.583	1.13 (0.73–1.75)	0.583	1.12 (0.73–1.74)	0.604
rs2228000 (HWE = 0.988)							
CC	111 (43.87)	205 (38.61)		1.00		1.00	
CT	108 (42.69)	250 (47.08)		0.80 (0.58–1.10)	0.170	0.80 (0.58–1.10)	0.166
TT	34 (13.44)	76 (14.31)		0.83 (0.52–1.32)	0.422	0.84 (0.52–1.33)	0.453
Additive			0.368	0.88 (0.71–1.09)	0.244	0.88 (0.71–1.10)	0.257
Dominant	142 (56.13)	326 (61.39)	0.160	0.80 (0.59–1.09)	0.160	0.81 (0.59–1.09)	0.162
Recessive	219 (86.56)	455 (85.69)	0.742	0.93 (0.60–1.44)	0.742	0.94 (0.61–1.46)	0.785
rs2229090 (HWE = 0.994)							
GG	99 (39.13)	191 (35.97)		1.00		1.00	
GC	105 (41.50)	255 (48.02)		0.79 (0.57–1.11)	0.175	0.79 (0.57–1.10)	0.169
CC	49 (19.37)	85 (16.01)		1.11 (0.73–1.71)	0.626	1.11 (0.73–1.71)	0.622
Additive			0.204	1.00 (0.81–1.24)	0.971	1.00 (0.81–1.24)	0.972
Dominant	154 (60.87)	340 (64.03)	0.392	0.87 (0.64–1.19)	0.392	0.87 (0.64–1.19)	0.384
Recessive	204 (80.63)	446 (83.99)	0.243	1.26 (0.85–1.86)	0.243	1.26 (0.86–1.87)	0.237

^a^ *χ* ^2^ test for genotype distributions between neuroblastoma cases and cancer-free controls.

^b^Adjusted for age and gender.

**Table 2:** Stratification analysis ofrisk genotypes and neuroblastoma susceptibility.

Variables	rs2228001 (cases/controls)		Adjusted OR	*P*	rs2228000 (cases/controls)		Adjusted OR^a^	*P* ^a^	rs2229090 (cases/controls)		Adjusted OR^a^	*P* ^a^
	AA	AC/CC	(95% CI)		CC	CT/TT	(95% CI)		GG	GC/CC	(95% CI)	
Age, month												
≤18	47/102	53/131	0.87 (0.54–1.39)	0.557	17/37	83/196	0.93 (0.49–1.74)	0.811	43/79	57/154	0.68 (0.42–1.10)	0.119
>18	52/116	101/182	1.24 (0.82–1.86)	0.304	17/39	136/259	1.20 (0.65–2.20)	0.559	56/112	97/186	1.04 (0.69–1.55)	0.862
Sex												
Females	46/95	55/138	0.81 (0.50–1.30)	0.383	17/31	84/202	0.75 (0.39–1.43)	0.382	37/89	64/144	1.08 (0.66–1.74)	0.771
Males	53/123	99/175	1.31 (0.87–1.97)	0.190	17/45	135/253	1.40 (0.77–2.55)	0.270	62/102	90/196	0.75 (0.50–1.13)	0.167
Sites of origin												
Adrenal gland	18/218	28/313	1.04 (0.56–1.94)	0.896	4/76	42/455	1.68 (0.58–4.85)	0.335	18/191	28/340	0.88 (0.47–1.64)	0.689
Retroperitoneal	37/218	48/313	0.90 (0.57–1.43)	0.654	14/76	71/455	0.84 (0.45–1.57)	0.582	36/191	49/340	0.75 (0.47–1.20)	0.226
Mediastinum	30/218	59/313	1.38 (0.86–2.22)	0.181	12/76	77/455	1.08 (0.56–2.08)	0.825	35/191	54/340	0.87 (0.55–1.38)	0.546
Other sites	11/218	14/313	0.92 (0.41–2.07)	0.837	3/76	33/455	1.31 (0.38–4.50)	0.670	8/191	17/340	1.16 (0.49–2.75)	0.734
Clinical stages												
I + II + 4s	50/218	73/313	1.03 (0.69–1.54)	0.872	15/76	108/455	1.24 (0.68–2.24)	0.486	51/191	72/340	0.79 (0.53–1.18)	0.257
III + IV	46/218	75/313	1.09 (0.72–1.64)	0.680	17/76	104/455	0.95 (0.54–1.69)	0.863	47/191	74/340	0.90 (0.60–1.35)	0.599

^a^Adjusted for age and gender.

## Abbreviations

GWAS: Genome-wide association study
SNP: Single nucleotide polymorphism
*XPC*: Xeroderma pigmentosum complementation C
NER: Nucleotide excision repair pathway
XP: Xeroderma pigmentosum
OR: Odds ratio
CI: Confidence interval.
