# Supplementary material for: Lack of Associations between XPC Gene Polymorphisms and Neuroblastoma Susceptibility in a Chinese Population
Source: Biomed Res Int. 2016 Oct 26;2016:2932049. doi: 10.1155/2016/2932049 (PMC5101359; doi:10.1155/2016/2932049)
Supplement: Supplementary file 1 — Supplemental Table 1: Frequency distribution of selected characteristics for neuroblastoma cases and cancer-free controls. [file 2932049.f1.doc]

| **Supplemental Table 1**: Frequency distribution of selected characteristics for neuroblastoma cases and cancer-free controls. | | | | | |
| --- | --- | --- | --- | --- | --- |
| Variables | Cases (n=256) | | Controls (n=531) | | *P^a^* |
|  | No. | % | No. | % |  |
| Age range, month | 0-156 | | 0.07-156 | | 0.239 |
| Mean ± SD | 30.87 ± 26.45 | | 29.73 ± 24.86 | |  |
| ≤18 | 101 | 39.45 | 233 | 43.88 |  |
| >18 | 155 | 60.55 | 298 | 56.12 |  |
| Gender |  |  |  |  | 0.333 |
| Female | 103 | 40.23 | 233 | 43.88 |  |
| Male | 153 | 59.77 | 298 | 56.12 |  |
| Clinical stages |  |  |  |  |  |
| I | 54 | 21.09 |  |  |  |
| II | 65 | 25.39 |  |  |  |
| III | 44 | 17.19 |  |  |  |
| IV | 77 | 30.08 |  |  |  |
| 4s | 9 | 3.52 |  |  |  |
| NA | 7 | 2.73 |  |  |  |
| Sites of origin |  |  |  |  |  |
| Adrenal gland | 46 | 17.97 |  |  |  |
| Retroperitoneal region | 87 | 33.98 |  |  |  |
| Mediastinum | 90 | 35.16 |  |  |  |
| Other region | 25 | 9.77 |  |  |  |
| NA | 8 | 3.13 |  |  |  |
| ^a^ Two-sided *χ^2^* test for distributions between neuroblastoma cases and cancer-free controls. | | | | | |
